# Evaluation of Colorectal Cancer Incidence Trends in the United States (2000–2014)

**DOI:** 10.3390/jcm7020022

**Published:** 2018-01-30

**Authors:** Benjamin E. Ansa, Steven S. Coughlin, Ernest Alema-Mensah, Selina A. Smith

**Affiliations:** 1 Institute of Public and Preventive Health, Augusta University, Augusta, GA 30912, USA; 2 Department of Clinical and Digital Health Sciences, College of Allied Health Sciences, Augusta University, Augusta, GA 30912, USA; scoughlin@augusta.edu; 3 Department of Community Health and Preventive Medicine, Morehouse School of Medicine, Atlanta, GA 30310, USA; eamensah@msm.edu; 4 Department of Family Medicine, Medical College of Georgia, Augusta University, Augusta, GA 30912, USA; sesmith@augusta.edu

**Keywords:** Colorectal cancer, incidence, rates, annual percent change, SEER

## Abstract

Colorectal cancer (CRC) incidence rates have declined in recent years for people of all races/ethnicities; however, the extent to which the decrease varies annually by demographic and disease-related characteristics is largely unknown. This study examines trends and annual percent change (APC) in the incidence among persons diagnosed with CRC in the United States of America from 2000–2014. The data obtained from the National Cancer Institute’s Surveillance, Epidemiology, and End Results (SEER) Program were analyzed, and all persons (*N* = 577,708) with malignant CRC recorded in the SEER 18 database from 2000 to 2014 were characterized according to sex, race, age at diagnosis, disease site and stage. Incidence rates and APC were calculated for the entire study period. Overall, the incidence rate of CRC decreased from 54.5 in 2000 to 38.6 per 100,000 in 2014, with APC = −2.66 (*p* < 0.0001). Decline in rates was most profound between 2008 and 2011 from 46.0 to 40.7 per 100,000 (APC = −4.04; *p* < 0.0001). Rates were higher for males (vs. females; rate ratio (RR) = 1.33) and for blacks (vs. whites; RR = 1.23). Proximal colon cancers at the localized stage were the predominant cancers. An increase in rate was observed among people younger than 50 years (6.6 per 100,000, APC= 1.5). The annual rate of CRC has decreased over time. However, the development and implementation of interventions that further reduce the disparities among demographic and disease-related subgroups are warranted.

## 1. Introduction

Colorectal cancer (CRC), a major clinical and public health concern, is the third most common cancer diagnosed and the second leading cause of cancer-related deaths for both men and women in the United States of America (US) [1]. According to the American Cancer Society, 135,430 new cases, and 50,260 deaths from CRC are expected to occur in 2017, and the lifetime risk of developing CRC is about 1 in 21 (4.7%) for men and 1 in 23 (4.4%) for women [2]. The management of CRC is associated with substantial health care costs, with national expenditures exceeding $14 billion annually [3,4].

The risk of CRC can be reduced through lifestyle modifications and screening [2,5]. Lifestyle factors that may reduce the risk of CRC include consuming a diet high in fruits and vegetables; maintaining a healthy body weight, minimizing alcohol intake; abstaining from tobacco use; use of nonsteroidal anti-inflammatory drugs; and greater consumption of calcium, fiber, folate, and vitamin D; and engaging in regular physical activity (colon cancer). Factors associated with an increased risk of CRC include older age, male sex, a family history of colon or rectal cancer, a history of colorectal polyps, inflammatory bowel disease, obesity, type 2 diabetes mellitus, and consuming a diet high in red and processed meat [2,5,6,7].

Since more than 90% of cases occur in people who are 50 years or older [5], the US Preventive Services Task Force recommends screening for CRC in adults beginning at age 50 years and continuing until age 75 [8]. Screening with both the noninvasive fecal occult blood test and colonoscopy reduce CRC incidence and mortality [8,9,10,11]. As a result of screening and improved treatment over the last few decades, there are now, in the US, more than 1 million survivors of CRC [2].

Factors such as race/ethnicity, age, socioeconomic status [12,13,14,15,16,17,18], and cancer-related characteristics influence CRC outcomes [19]. Although, in recent years, CRC incidence and mortality rates have declined for men and women of all races/ethnicities [1], the extent to which the decrease varies annually by age, race, gender, site and stage is largely unknown. To better inform future research on CRC, and to guide the planning and implementation of programs for CRC prevention and control, the present study of current data for the CRC population in the US examines trends and annual percent change (APC) in the incidence among persons diagnosed with CRC from 2000–2014.

## 2. Materials and Methods

### 2.1. Data Source

The data were obtained from the National Cancer Institute’s Surveillance, Epidemiology, and End Results (SEER) Program, which is a main source for cancer statistics in the US and includes information on incidence, prevalence, and survival from specific geographic areas representing 28% of the population [20,21]. SEER also compiles reports on cancer mortality for the entire country [20,21]. Data collection began in the early 1970s and gradually expanded. Data for the present analysis were obtained from the 18 SEER registries that are currently operating.

### 2.2. Study Population and Measures 

All persons with CRC recorded in the SEER 18 database from 2000 to 2014 were characterized according to sex, race, age at diagnosis, and disease site and stage. Included were cases in the SEER database with malignant behavior and known age; cases of unknown age were excluded. Sex was reported as either male or female; race was classified as white, black, other (American Indian, Alaskan Native, Asian/Pacific Islander), or unknown. Ages were grouped as <40, 40–49, 50–59, 60–69, 70–79, and 80+ years.

CRCs were grouped into three major anatomic primary sites: proximal colon, distal colon, and rectum. The International Classification of Diseases for Oncology, third edition (ICD-O-3) was used to select all CRC malignant cases reported to the SEER Program from 2000 through 2014 that originated from the following primary sites (and ICD-O-3 codes): proximal colon (cecum (C18.0), appendix (C18.1), ascending colon (C18.2), hepatic flexure of colon (C18.3), transverse colon (C18.4), splenic flexure of colon (C18.5)); distal colon (descending colon (C18.6), and sigmoid colon (C18.7)); and rectum (recto-sigmoid junction (C19.9), and rectum, not otherwise specified (C20.9)). Overlapping lesion of colon (C18.8), and colon, not otherwise specified (C18.9) were grouped as other.

Stage at diagnosis was categorized according to the SEER historic stage A classification scheme. It categorizes cancer cases as localized, regional, distant, or unstaged based on the following definitions [20,21]: localized cancer is cancer that is limited to the organ in which it began without evidence of spread; regional cancer is cancer that has spread beyond the original (primary) site to nearby lymph nodes or organs and tissues; distant cancer is cancer that has spread from the primary site to distant organs or distant lymph nodes; and unstaged cancer is cancer for which there is not enough information to indicate a stage.

### 2.3. Statistical Analyses

For the entire study period, incidence rates and trends of CRC were calculated by sex, race, age, stage and site, and were expressed as the number of cases per 100,000 individuals (age-adjusted to the 2000 US Standard Population) accompanied by the 95% confidence intervals (CIs). SEER*Stat software (version 8.3.4, Surveillance Research Program, National Cancer Institute, Bethesda, MD, USA) [22] was used for analyzing incidence rates, trends, and rate ratios. Rate ratios (RRs) and the corresponding 95% CIs were calculated, as described by Tiwari et al. [23], to examine the differences in rates between men and women; among whites, black, and individuals of other racial groups; between the age groups; and among the sites and stages. 

Changes in incidence rates over time were evaluated and expressed as APC and corresponding CIs and *p*-values. This was calculated by utilizing Joinpoint Regression Program (Version 4.5.0.1, Statistical Methodology and Applications Branch, Surveillance Research Program, National Cancer Institute, Bethesda, MD, USA) [24,25,26]. The APC indicates the cancer rate change at a constant percentage of the rate of the previous year, and is obtained by fitting a least-squares regression line to the natural logarithm of the rates using the calendar year as a regressor variable (the model is linear on the log of the response for calculating annual percentage rate change) [21,25,27]. The incidence data were further analyzed by use of joinpoint models, which were aimed at evaluating longitudinal data for a change in trend. An APC was computed for each of those trends by means of generalized linear models, assuming a Poisson distribution. Changes in trend were tested for statistical significance using a Monte Carlo permutation method [25,26]. All *p*-values for significance testing of APC = 0 were two-sided, and considered to be of statistical significance when *p* < 0.05 [27]. Significant changes included changes in direction or in the rate of increase or decrease.

## 3. Results

### 3.1. Demographic and Cancer Characteristics

Between 2000 and 2014, a total of 577,708 malignant CRC cases were recorded in the 18 SEER registries (Table 1). The majority were male (51.5%, *n* = 297,320), predominantly white (80%, *n* = 462,221), and older than 50 years (90%, *n* = 519,770). Almost 43% (*n* = 247,656) presented with cancer in the proximal colon that was localized (41%, *n* = 236,262). The distributions of patient demographic characteristics and cancer primary sites did not differ significantly over time.

### 3.2. CRC Rates/Trends for each Year from 2000 to 2014

**Figure 1:**
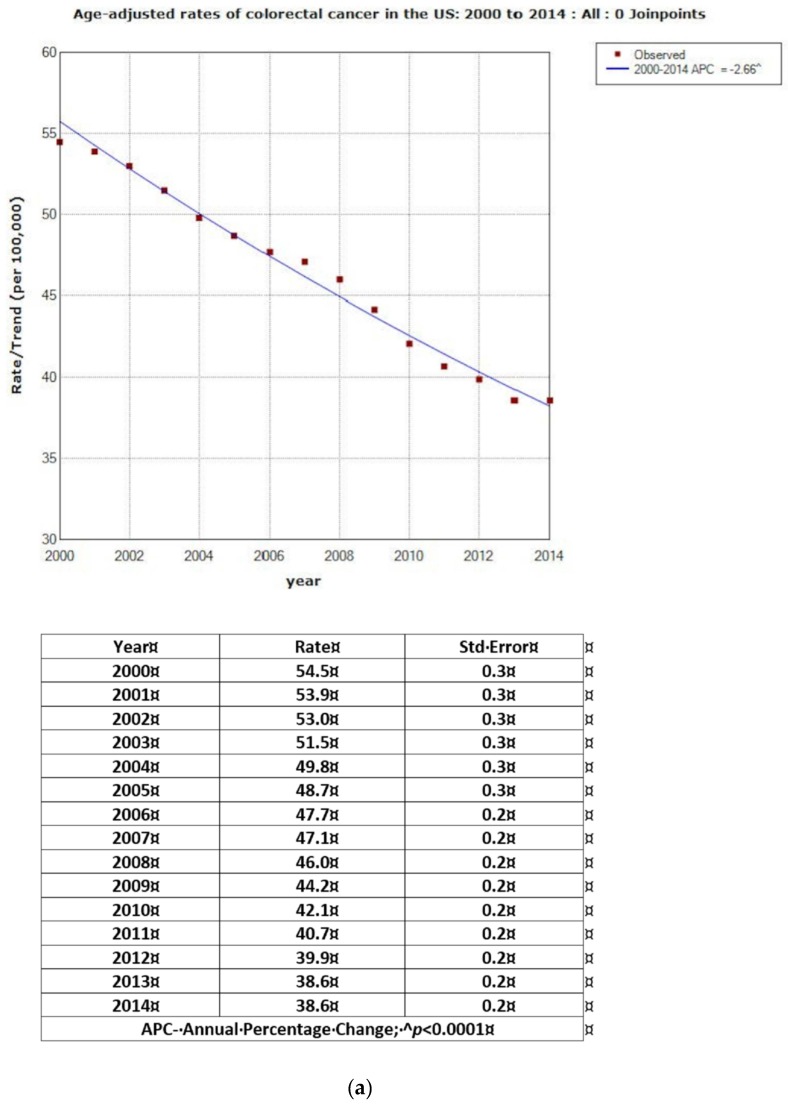

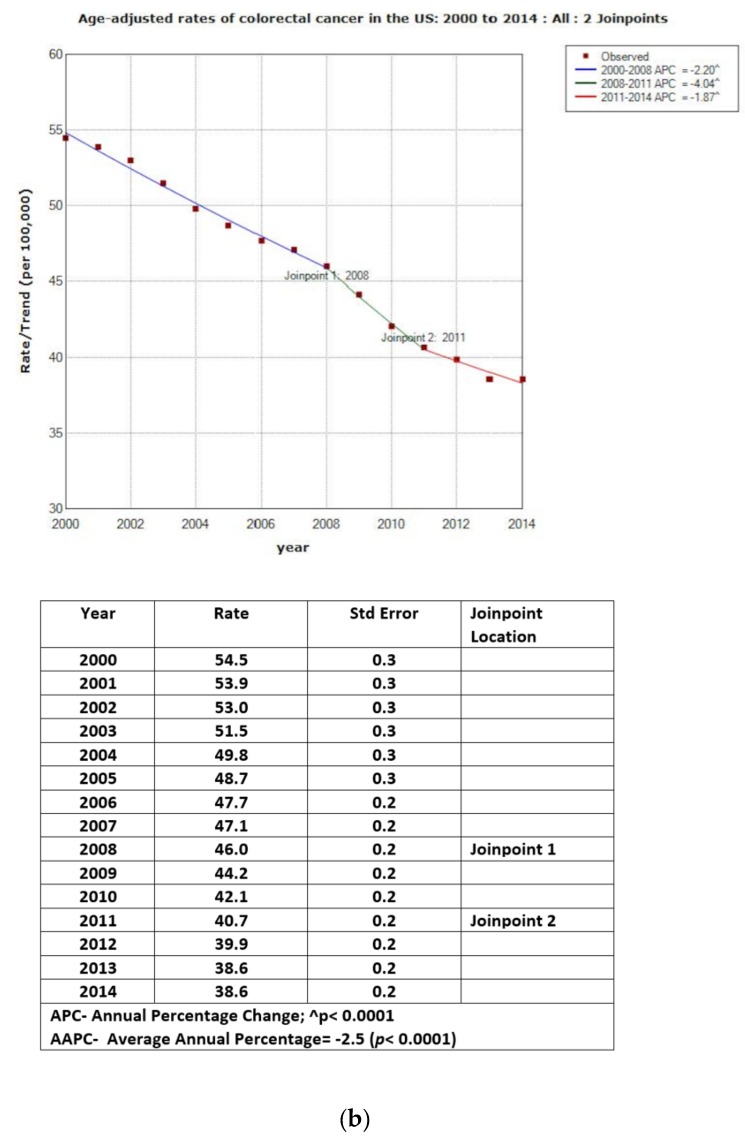
(**a**) Age-adjusted rates of colorectal cancer in USA: 2000–2014: All: 0 Joinpoints. (**b**) Age-adjusted rates of colorectal cancer in USA: 2000 to 2014: All: 2 Joinpoints.

**Figure 2:**
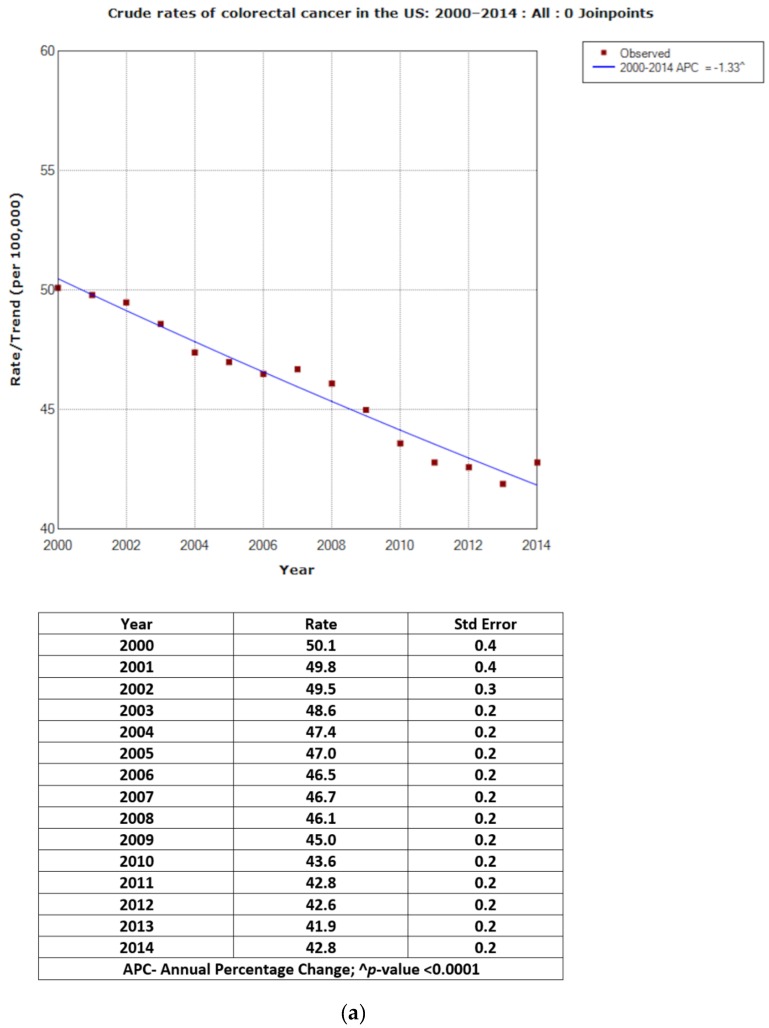

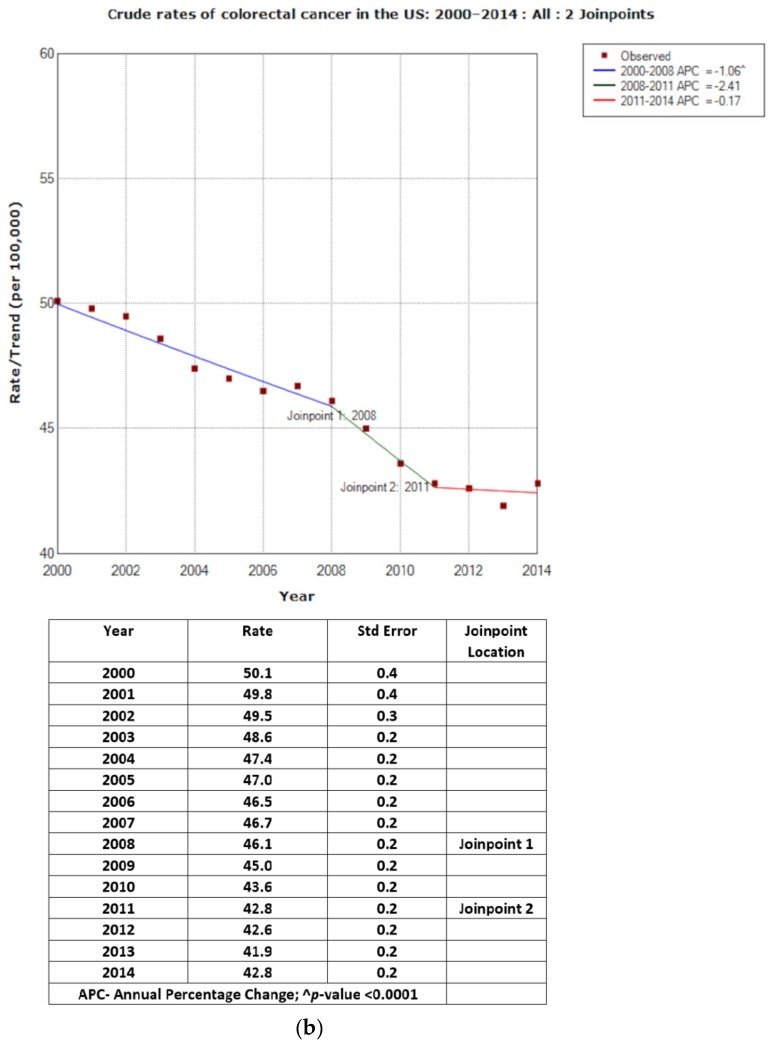
(**a**) Crude rates of colorectal cancer in the USA: 2000–2014: All: 0 Joinpoints. (**b**) Crude rates of colorectal cancer in the USA: 2000–2014: All: 2 Joinpoints.

Figure 1a shows the age-adjusted rates and APC for CRC cases. Overall, the rate of CRC decreased from 54.5 in 2000 to 38.6 per 100,000 in 2014, with APC = −2.66 (*p* < 0.0001). Figure 1b illustrates two joinpoints for rates and corresponding APCs. Between 2000 and 2008, rates decreased from 54.5 to 46.0 per 100,000 (APC = −2.20; *p* < 0.0001); and, between 2008 and 2011, from 46.0 to 40.7 per 100,000 (APC = −4.04; *p* < 0.0001). Between 2011 and 2014, the decrease from 40.7 to 38.6 per 100,000 was statistically significant (APC = −1.87; *p* < 0.0001). The overall crude rates were slightly lower with the overall APC = −1.33; *p* < 0.0001 (zero joinpoint). The two joinpoints for the crude rates showed corresponding lower APCs for 2000–2008, 2008–2011 and 2011–2014 (−1.06, −2.41 and −0.17 respectively) (Figure 2a,b).

### 3.3. Overall CRC Rates/Trends for the Entire Time Period between 2000 and 2014

As presented in Table 2, the overall age-adjusted incidence rate of CRC in the US for all individuals and all ages during the study period was 45.9 per 100,000 (95% CI = 45.8, 46.0 per 100,000), with APC = −2.7 (*p* < 0.0001). Although persons who were 60 years and older had higher rates of CRC, the decline in CRC incidence was also greater in this population (APCs between −3.3 and −3.8 per 100,000) compared to persons who were 50–59 years old (APCs = −0.5). An increasing trend was observed among persons less than 50 years, with rates at 2.3 per 100,000 (APC = 2.7; *p* < 0.0001) for persons <40 years, and 22.5 per 100,000 (APC = 1.7; *p* < 0.001) for those between 40 and 49 years. The incidence rate was higher for blacks (56.1 compared to 45.6 per 100,000 for whites), but the APC was slightly greater for whites than for blacks (−2.7 vs. −2.5). Males had a higher incidence rate and greater APC (53.4 per 100,000 (APC = −2.9; *p* < 0.001)) than females (39.9 per 100,000 (APC = −2.5; p < 0.0001)). More people presented with cancers of the proximal colon (20.0 per 100,000) that were localized (18.7 per 100,000). The greatest declines in CRC rates were among persons 70 years and older (APC = −3.8; *p* < 0.0001) and among those presenting with distal colon cancer (APC = −3.6; *p* < 0.0001). The highest APCs for declining rates were in cases of regional spread (APC= -3.3; *p* < 0.0001) and unstaged cancer (APC = −3.5; *p* < 0.0001). The crude rate ratios for the demographic and disease-related variables were only slightly lower from the age-adjusted values (results not shown).

## 4. Discussion

This study provides important information about the incidence and trends of CRC in the US. The study population consisted of slightly more males, was mostly whites, and was 50 years and older. The overall incidence rate for CRC between 2000 and 2014 was 45.9 per 100,000 and APC = −2.7. Rates were higher for males (vs. females; RR = 1.33) and for the blacks (vs. whites; RR = 1.23). Proximal colon cancers at the localized stage were the predominant cancers. The greatest decrease in rates was for cancers in the distal colon, at localized stage, and among persons older than 70 years. There was an increase in rate among people younger than 50 years (6.6 per 100,000, APC = 1.5).

The current results are similar to those reported by previous studies that analyzed temporal trends in CRC incidence [28,29,30,31,32]. Siegel et al. [28], analyzed the SEER 9 data for 2009 through 2013, and reported an overall incidence rate for CRC of 40.7 per 100,000 persons. There was a 32% decline in the rate among people 50 years and older and an accelerated rate of decline, from 298.3 (per 100,000) in 2000 to 186.8 in 2013, for people 65 years and older. From 2000 to 2013, CRC incidence rates increased by 22% among individuals younger than 50 years. Incidence rates were higher for men than for women and were higher for blacks than for whites and other racial groups [29,31,32]. The most common tumor location was the proximal colon (42%), followed by the rectum (28%) [28,30].

Increased uptake of screening, which rose from 38% in 2000 to 59% in 2013 for adults aged 50 years or older, may explain the declines in CRC incidence among age groups older than 55 years [33,34,35]. Differences in the uptake of screening may also account for differences in the incidence trends for individuals aged 50 years and older. The receipt of a colonoscopy in the past 10 years increased dramatically from 14% in 2000 to 41% in 2013 among individuals of ages 50 to 54 years, from 16% to 52% in those of ages 55 to 59 years, and from 25% to 63% in those of ages 65 years and older [33].

Lifestyle behaviors, such as consuming unhealthy diets and physical inactivity, with the resultant increase in the prevalence of overweight and obesity are possible factors contributing to the increased rates of CRC among people younger than 50 years [34]. CRC in younger patients tends to be more aggressive and more advanced at presentation than late-onset CRC [16,36,37]. Adults younger than 50 years without risk factors are not recommended to undergo routine screening for CRC, and this may also be a potential explanation for the rising incidence trends, and advanced cancer presentation in this population [18,27]. The data from this study revealed an APC in the incidence of CRC of 2.7 and 1.7 for individuals <40 and 40–49 years respectively. These results are similar to those from Siegel et al [34] revealing that adults ages 20 to 39, had colon cancer incidence rates increase by 1% to 2% per year; and adults 40 to 54, with rates increase by 0.5% to 1% per year from the mid-1990s through 2013. Also, Bailey et al (2015) reported a 1.99 % increase in the rates of CRC among 20–34 year old, and 0.41% increase among 35–49 year old individuals from 1975–2010. These data give credence for the need to review the current guidelines for CRC screening to begin at earlier ages than those currently recommended.

The sharp decline observed in the overall rates (APC of −4.40) from 2008 through 2011 (Figure 1b) is likely due in part to increased uptake of routine CRC screening. CRC screening uptake in the US may have received a boost after being promoted at the nation’s first colon cancer awareness month in March of 2000 and public health campaigns such as Screen for Life [38,39,40]. The high rate of proximal colon cancer compared to the other subsites may be due to the use of flexible sigmoidoscopy with inspection of the rectum and distal colon. More evidence-based studies may further explain the higher rate of proximal colon cancer.

The strength of this study includes the large sample size which enables SEER-based studies to have sufficient power for detecting relatively moderate associations and permits a variety of multivariable analyses [21,41]. The population- based, as opposed to institution-based, identification of patients increases the generalizability of findings. Although institutional studies often gather more detailed information about each patient, those studies usually are confined to major referral centers and may not be representative of the patients with CRC who are treated in community hospitals and clinics. The main limitation of this study pertains to the lack of data on demographic variables, such as health insurance coverage, education, marital, and income status. SEER 18 registries contain data representing only 28% of the U.S.A. population that are not randomly selected; therefore, selection and confounding biases are possible limitations [42].

## 5. Conclusions

The annual rate of CRC has decreased over time, especially among individuals older than 70 years; however, not all population subgroups are benefitting from this trend. The development and implementation of interventions that further reduce the disparities in CRC incidence among demographic and disease-related subgroups are warranted.

## Figures and Tables

**Table 1:** Characteristics of colorectal cancer (CRC) cases by year of diagnosis (18 Surveillance, Epidemiology, and End Results (SEER) Program registries, 2000–2014).

Characteristics	Total *N* = 577,708 (%)	2000–2004 *n* = 197,716 (%)	2005–2009 *n* = 193,012 (%)	2010–2014 *n* = 186,980 (%)
Sex				
Male	297,320 (51.5)	100,633 (51.0)	99,304 (51.4)	97,383 (52.1)
Female	280,388 (48.5)	97,083 (49.1)	93,708 (48.6)	89,597 (47.9)
Race				
White	462,221 (80.0)	162,577 (82.2)	154,310 (79.9)	145,334 (77.7)
Black	66,475 (11.5)	21,399 (10.8)	22,327 (11.6)	22,749 (12.2)
* Other	44,989 (7.8)	12,950 (6.6)	15,171 (7.9)	16,868 (9.0)
Unknown	4023 (0.7)	790 (0.4)	1204 (0.6)	2029 (1.1)
Age				
<40	15,312 (2.6)	4631 (2.3)	4944 (2.6)	5737 (3.1)
40–49	42,626 (7.4)	13,174 (6.7)	14,556 (7.5)	14,896 (8.0)
50–59	103,684 (18.0)	30,620 (15.5)	35,399 (18.3)	37,665 (20.1)
60–69	131,512 (22.8)	42,519 (21.5)	43,316 (22.4)	45,677 (24.4)
70–79	148,149 (25.6)	57,591 (29.1)	48,458 (25.1)	42,100 (22.5)
80+	136,425 (23.6)	49,181 (24.9)	46,339 (24.0)	40,905 (22.0)
Stage				
Localized	236,262 (40.9)	80,112 (40.5)	80,050 (41.5)	76,100 (40.7)
Regional	192,289 (33.3)	68,430 (34.6)	63,916 (33.1)	59,943 (32.0)
Distant	111,909 (19.4)	35,994 (18.2)	36,878 (19.1)	39,037 (20.9)
Unstaged	37,248 (6.4)	13,180 (6.7)	12,168 (6.3)	11,900 (6.4)
** Primary site				
Proximal colon	247,656 (42.9)	84,430 (42.7)	83,209 (43.1)	80,017 (42.8)
Distal colon	138,377 (23.9)	49,673 (25.1)	46,207 (23.9)	42,497 (22.7)
Rectum	165,932 (28.7)	54,840 (27.7)	55,177 (28.6)	55,915 (29.9)
*** Other	25,743 (4.5)	8,773 (4.5)	8,419 (4.4)	8,551 (4.6)

Note: SEER- Surveillance, Epidemiology, and End Results; * Other race- American Indian/AK Native, Asian/Pacific Islander; ** Primary site: proximal colon-cecum, appendix, ascending colon, hepatic flexure, transverse colon, splenic flexure; distal colon-descending colon, sigmoid colon; rectum- rectosigmoid junction, rectum not otherwise specified; *** Other site-overlapping lesion of colon, and colon, not otherwise specified.

**Table 2:** Age-adjusted rates of CRC in the US, (18 Surveillance, Epidemiology, and End Results (SEER) Program registries, 2000–2014).

Population Groups	Rate	Standard Error	95% C.I (Rate) Lower/Upper		Rate Ratio	95% C.I (Rate Ratio) Lower/Upper		APC	*p*-Value (APC)
All groups	45.9	0.1	45.8	46.0				−2.7	<0.001
Sex									
Male	53.4	0.1	53.2	53.6	Ref.			−2.9	<0.001
Female	39.9	0.1	39.8	40.1	0.75	0.74	0.75	−2.5	<0.001
Race									
White	45.6	0.1	45.5	45.7	Ref.			−2.7	<0.001
Black	56.1	0.2	55.6	56.5	1.23	1.22	1.24	−2.5	<0.001
* Other	37.5	0.2	37.2	37.9	0.82	0.81	0.83	−2.3	<0.001
Age (Years)									
<40	2.3	0.0	2.3	2.4	Ref.			2.7	<0.001
40–49	22.5	0.2	22.1	22.8	9.72	9.41	10.03	1.7	<0.001
50–59	64.1	0.2	63.7	64.5	27.27	9.67	9.87	−0.5	<0.001
60–69	128.6	0.4	127.9	129.3	53.55	19.41	19.80	−3.3	<0.001
70–79	232.2	0.6	231.0	233.4	100.45	35.07	35.75	−3.8	<0.001
80+	329.8	0.9	328.1	331.6	142.98	49.81	50.79	−3.8	<0.001
Stage									
Localized	18.7	0.0	18.7	18.8	Ref.			−2.6	<0.001
Regional	15.4	0.0	15.3	15.4	0.82	0.82	0.83	−3.3	<0.001
Distant	8.9	0.0	8.8	8.9	0.47	0.47	0.48	−1.3	<0.001
Unstaged	2.9	0.0	2.9	3.0	0.16	0.15	0.16	−3.5	<0.001
** Primary site									
Proximal colon	20.0	0.0	19.9	20.1	Ref.			−2.5	<0.001
Distal colon	10.9	0.0	10.9	11.0	0.55	0.54	0.55	−3.6	<0.001
Rectum	13.0	0.0	12.9	13.0	0.65	0.64	0.65	−2.0	<0.001
*** Other	2.1	0.0	2.0	2.1	0.10	0.10	0.10	−2.5	<0.001

Note: SEER- Surveillance, Epidemiology, and End Results; rates are per 100,000 and age-adjusted to the 2000 US standard population (19 age groups—Census P25-1130); C.I- confidence intervals (Tiwari model) are 95% for rates and ratios; APC is significantly different from zero if *p* < 0.05, * Other race- American Indian/AK Native, Asian/Pacific Islander, ** Primary site: proximal colon- cecum, appendix, ascending colon, hepatic flexure, transverse colon, splenic flexure; distal colon- descending colon, sigmoid colon; rectum- rectosigmoid junction, rectum not otherwise specified; *** Other site- overlapping lesion of colon, and colon, not otherwise specified.
